# Hot and Cool Executive Function in Children with Autism Spectrum Disorder and Schizotypal Traits

**DOI:** 10.3390/brainsci15030282

**Published:** 2025-03-06

**Authors:** Evangelia Chrysanthi Kouklari, Evdokia Tagkouli, Vassiliki Ntre, Artemios Pehlivanidis, Stella Tsermentseli, Nikos C. Stefanis, Chris Pantelis, Katerina Papanikolaou

**Affiliations:** 1 Department of Child Psychiatry, Aghia Sophia Children’s Hospital, National and Kapodistrian University of Athens, 115 27 Athens, Greece; 2 1st Department of Psychiatry, Eginition Hospital, National and Kapodistrian University of Athens, 115 28 Athens, Greece; 3 Department of Primary Education, University of Thessaly, 382 21 Volos, Greece; 4 Department of Psychiatry, The University of Melbourne, Parkville, VIC 3010, Australia; 5 Florey Institute of Neuroscience and Mental Health, The University of Melbourne, Parkville, VIC 3010, Australia; 6 Monash Institute of Pharmaceutical Sciences (MIPS), Monash University, Royal Parade, Parkville, VIC 3052, Australia

**Keywords:** ASD, executive function, schizotypal traits

## Abstract

**Background:** Schizotypal traits are notably prevalent among children diagnosed with Autism spectrum disorder (ASD). Both conditions commonly exhibit impairments in executive functions (EF), which encompass cool and hot processes. The observed deficits in these EF domains across ASD and schizotypy underscore a compelling need to investigate how their co-occurrence impacts EF. **Methods:** This study investigated the impact of co-occurring autistic and schizotypal traits on EF in 63 children diagnosed with ASD, aged 7 to 12 years. Participants were assessed using the Autism Diagnostic Observation Schedule-2 (ADOS-2), the Melbourne Assessment of Schizotypy in Kids (MASK), and a battery of hot and cool EF tests. **Results:** Correlational analyses revealed a significant association between MASK score and working memory, as well as between ADOS scores and various cool EF components (i.e., working memory, inhibition and planning). Hierarchical regression analyses showed that the interaction between ADOS and MASK scores significantly predicted performance on hot EF (i.e., affective decision-making), but not on cool EF tasks. **Conclusions:** These findings suggest that the co-occurrence of ASD and schizotypal traits may have differential effects on cool and hot EF domains. Understanding how the combination of autistic and schizotypal traits affects cognitive processes may inform tailored interventions and support strategies for individuals presenting with these traits.

## 1. Introduction

Autism spectrum disorder (ASD) and schizotypal personality disorder (SPD) are distinct complex conditions (Diagnostic and Statistical Manual of Mental Disorders [DSM]-5, [1]) that have been extensively studied in isolation. ASD is characterized by neurodevelopmental impairments in social interactions, verbal/non-verbal communication, and repetitive behaviors/interests. SPD is a nonpsychotic condition within the schizophrenia spectrum, presenting milder schizophrenia-related symptoms and diagnosable in children as young as 6 years old [2,3,4]. SPD display cognitive and perceptual distortions, manifesting in peculiar behaviors, bizarre ideation, and a fixation on self-referential thoughts and fantastical events [5,6]. While SPD has traditionally been considered an adult diagnosis, the DSM-5 has formally recognized that schizotypal disorder can manifest in childhood, categorizing it within the Schizophrenia Spectrum Disorders (SSD) [7]. However, schizotypal traits exist on a continuum, and not all children displaying these characteristics will meet full diagnostic criteria for schizotypal disorder. Dimensional approaches to schizotypy emphasize the assessment of traits rather than categorical diagnosis, allowing for a more refined understanding of early schizotypal tendencies as potential developmental markers (e.g., [8]).

The clear demarcation between ASD and SPD remains uncertain [9], especially given accumulating evidence suggesting shared etiological and risk factors between them, resulting in co-occurrence at both diagnostic and symptom/trait levels (e.g., [10,11]). Schizotypal symptoms manifest significantly in children with ASD and vice versa [3,12]. For instance, previous evidence (e.g., [13]) has indicated that 41% of adolescents diagnosed with ASD fulfilled the criteria for SPD (as outlined in DSM-IV-TR). Negative symptoms, and to a lesser degree, disorganized symptoms, of SPD have been documented in both adults and adolescents diagnosed with ASD (e.g., [14,15]). Conversely, the presence of positive symptoms has not been exhibited consistently among individuals with ASD (e.g., [16,17]). This underscores the need to explore the nature of their association and the impact of their concurrent presence on children’s phenotype and functional outcomes.

Dysfunction in the frontal lobe characterizes both schizophrenia spectrum (including schizotypy) and ASD (e.g., [18,19,20]). Impairments in the frontal lobe are linked to disruptions in higher-order cognitive processes, namely, executive functions (EF) (see [21] for a review). EF is a set of future oriented, goal-directed cognitive processes such as working memory, planning, cognitive flexibility, and inhibition [22]. EFs enable individuals to consciously regulate behavior, emotions, and thoughts. It is contended that EF abilities vary—as a function of the emotional/motivational significance of the task—from hot–affective EF to cool–cognitive EF [23,24]. Cool EF aspects (i.e., working memory, cognitive flexibility, inhibition, and planning) are invoked within abstract, decontextualized, and largely emotionally neutral situations, with regulatory functions attributed to lateral sectors of the prefrontal cortex (specifically, dorsolateral and ventrolateral regions), as posited by extant research (e.g., [25]). Conversely, hot EF (i.e., affective decision-making, delay discounting/of gratification) pertains to processes requisite in situations imbued with motivational/reward and emotional significance, relying upon the orbitofrontal cortex and ventromedial prefrontal cortex areas (e.g., [25]). Notably, these cerebral locales have been observed to interface with the limbic system, particularly the amygdala, which underpins emotional processing [26]. It is noteworthy that hot EF tasks entail meaningful outcomes, whether rewards or losses, for the individuals. While cool EF processes diverge from hot, they are postulated to collaborate as constituents of a broader functional construct, contingent upon the exigencies of each task [23].

Extensive deficits in various cool EF domains (such as inhibition, working memory, cognitive flexibility, and planning) have been observed across the lifespan in ASD (for a meta-analysis, see [27,28]). Previous studies investigating the correlation between EF (e.g., inhibition, working memory, planning/organization, cognitive flexibility) and symptoms of ASD, assessed through either ADOS or ADI, have presented convincing findings indicating a link between EF and autistic symptomatology (e.g., [29,30,31,32,33]). Similarly, a comprehensive review of cognition in schizotypy [18] indicated deficits in inhibitory control, selective and sustained attention, incidental learning, and memory in higher schizotypy. Studies examining EF abilities solely in schizotypy have reported that individuals with higher levels of schizotypal traits demonstrate impairments in both verbal and visual–spatial working memory, cognitive flexibility, and response inhibition (e.g., [34]). Moreover, EF shifting has been shown to be notably linked with schizotypy, particularly in relation to dimensions such as introvertive anhedonia, cognitive disorganization, and unusual experiences (e.g., [35]).

In the realm of hot EF, studies have consistently revealed deficits in affective decision-making (e.g., [36,37,38]) as well as delays of gratification/discounting in ASD (e.g., [39,40,41]). Similarly, research within the schizophrenia spectrum has indicated compromised performance in affective decision-making (e.g., [42,43,44]), alongside findings suggesting that individuals exhibiting elevated negative schizotypy manifest distinct neural responses during reward anticipation [45] compared to controls. Moreover, a notably higher rate of delay discounting has been observed in schizophrenia and schizotypy relative to controls in delay discounting tasks [46,47,48].

Given the extensive deficits identified above in both cool and hot EF domains in ASD and schizotypy, there emerges a compelling need to investigate the impacts of their co-occurrence on EF. While EF deficits have been reported separately for ASD and schizotypy, direct investigation into how their co-occurrence affects EF in children remains limited. The examination of the combined influence of ASD and schizotypy on EF may hold promise in shedding light on the complex interplay between these neurodevelopmental and psychotic conditions, potentially informing more effective interventions and treatments tailored to individuals presenting with both conditions. Surprisingly, previous investigations have proposed that concurrent manifestations of autistic and positive schizotypal traits may exert a moderating influence on behavior and cognition. More specifically, previous research [49] demonstrated the contrasting impacts of co-occurring autistic and positive schizotypal traits on mentalizing difficulties (understand the perspective of others), suggesting a potential ameliorative effect on such challenges due to the interaction between autistic and schizotypal traits. Moreover, the co-occurrence of ASD and schizotypal traits has been shown to reduce the cost incurred in the presence of salient distractors [50]. Furthermore, a study [51] found distinct associations of attentional networks with autistic and schizotypal traits in college students. Autistic traits were linked to the orienting network, while schizotypal traits were associated with both the executive control and orienting networks.

With regard to EF, a study [52] examined the co-occurrence of autistic and schizotypal traits in college students, and found that their interaction was associated with improved EF. Finally, previous research [2] found that a comorbid group (ASD with schizotypy) presented better performance on an intra-/extra-dimensional task (attentional set-shifting) compared to both the sole ASD and schizotypy groups. It should be noted though that this finding was particularly relevant to this task, and was not demonstrated for the other EF tasks addressed in that study. This indicates that the improved performance of the comorbid ASD and schizotypy group was particular to attentional set-shifting, and cannot be generalized to better overall EF. As evidenced by this limited research, the examination of the co-occurrence of autistic and schizotypal symptoms has focused only on cool EF. Consequently, the potential moderating effects of co-occurring autistic and schizotypal traits on both hot and cool EF remain unclear.

In the current study, we aim to address this gap in the existing ASD and schizotypy literature by simultaneously assessing autistic and schizotypal traits in a cohort of children diagnosed with ASD, aiming to elucidate the impact of their co-existence on hot and cool EF. By focusing on a developmental population, this study provides a novel contribution to understanding how neurodevelopmental and schizophrenia spectrum traits interact in shaping EF outcomes.

### Objectives

Our main objective was to examine the impact of the co-occurrence of ASD and schizotypal traits on EF in children diagnosed with ASD. We aimed to investigate this relationship including both cool and hot EF aspects, as the role of autistic and/or schizotypal traits in EF has been examined through a purely cognitive lens (i.e., cool EF processes). Based on previous studies (e.g., [2]), we hypothesized that the co-occurrence of ASD and schizotypal symptoms in children would be associated with higher EF performance.

## 2. Materials and Methods

### 2.1. Participants

The sample consisted of 63 children diagnosed with ASD, ranging in age from 7 to 12 years. Participants were recruited through the Child and Adolescent Psychiatry ASD outpatient clinic (Special ASD clinic) at the Department of Child Psychiatry in Greece’s largest children’s hospital (see Table 1 for sample’s characteristics). Inspection of their records confirmed that all participants were considered high-functioning and had received an official ASD diagnosis based on DSM-5 criteria [1]. Participants with comorbid disorders (e.g., attention deficit/hyperactivity disorder, other psychiatric and neurological disorders), those currently taking psychotropic and antiepileptic medication, those with color blindness, or those with a Full-Scale Intelligence Quotient (FSIQ) below 70 were excluded from the study. The research received ethical approval from the hospital’s ethics board, and written informed consent was obtained from all participants’ parents/guardians.

### 2.2. Neuropsychological Measures

#### 2.2.1. Cool EF

Stroop Color and Word Test [53]. The Stroop test evaluates individuals’ capacity to suppress cognitive interference, wherein the processing of one stimulus conflicts with that of another simultaneous stimulus [54]. This assessment involves prompting participants to identify the color of ink in which different color names are printed, rather than reading the actual words. Notably, the color names are printed in inks of differing colors, necessitating respondents to identify the ink color while disregarding the word itself. Number of errors in identifying the ink color was recorded during the test administration. Widely utilized as a measure of color–word interference, the Stroop test demonstrates notable reliability across repeated assessments (e.g., [55]).

Berg’s Card-Sorting Task-64 (adapted from the PEBL Platform [56]). This test is a computerized assessment modeled after the Wisconsin Card Sorting Test. In this task, participants are instructed to categorize cards exhibiting various features into distinct piles, according to an undisclosed and variable rule. Each card must be sorted into one of four different piles, with feedback provided solely on the accuracy of the sorting. Participants have the option to categorize cards based on the color, shape, or number of symbols present. Notably, the sorting rule undergoes alteration every 10 cards, challenging participants’ adaptability and cognitive flexibility. The number of correctly sorted cards is recorded. Reliability assessments of computerized renditions of this task have consistently yielded coefficients exceeding 0.90 [57].

The Tower of London test [58] measures individuals’ planning abilities. The wooden boards used comprise three wooden beams, each supporting three balls of distinct colors—green, red, and blue. Participants are tasked with replicating a sequence of patterns using solely the wooden balls, adhering to a predetermined number of movements outlined by the researcher’s instructions. Following the provision of instructions, participants are presented with a 3-move problem for practice, after which they proceed to tackle 12 planning problems. Successful completion of each planning problem necessitates adherence to two primary rules: firstly, the completion of each problem within a specified number of moves, and secondly, the removal of only one ball per beam per move. Scoring is based on the number of problems successfully completed without violating these rules, with participants receiving one point for successful completion and zero points for failure. The Tower of London test stands as one of the most commonly employed measures of planning abilities across various life stages [59], exhibiting favorable test–retest reliability [60].

Forward and Backward Digit Span tests (Wechsler Intelligence Scale for Children Fifth Edition [61]). In this assessment of verbal working memory, individuals were required to recall and repeat sequences of random numbers in the exact same order as presented by the examiner. Notably, in the backward digit span subtest, participants were tasked with repeating the sequence of numbers in reverse order. The successful completion of two trials within a block enabled progression to subsequent ones. Scoring was based on the accuracy of responses, with one point awarded for each correctly recalled trial. Composite working memory scores were derived from the total sum of points achieved across both tests. The efficacy of digit span as a measure of working memory has been extensively researched, and is widely acknowledged for its high reliability and validity [62].

#### 2.2.2. Hot EF

Iowa Gambling Task (IGT [63]). This computerized version [56] measures affective decision-making. In this task, participants were presented with four distinct card categories labeled A, B, C, and D, and instructed to select cards using a mouse interface. Each card choice resulted in either monetary gain or loss, with some cards being advantageous (resulting in monetary gain) and others disadvantageous (resulting in monetary loss). Across 100 card selections, participants encountered varying degrees of potential gains and losses associated with each card category. Specifically, categories A and B were designed to be equivalent in terms of total net loss over a series of 20 card selections, while categories C and D were equivalent in total net winnings. Category A incurred frequent but smaller losses, whereas category B incurred less frequent but higher losses. Conversely, categories C and D offered potential gains, with category D having less frequent but higher-magnitude losses compared to category C. Participants’ affective decision-making tendencies were evaluated based on whether they predominantly made “advantageous” or “disadvantageous” choices throughout the task. Scoring followed a methodology similar to previous studies, such as [64], where scores were derived by subtracting the number of disadvantageous choices (categories A and B) from the number of advantageous choices (categories C and D), divided by the total number of trials (100). The reliability of the IGT has been established in prior research, with documented test–retest correlations ranging from r_s_ = 0.64 to 0.82 (e.g., [65]).

The Delay Discounting Task [66] involves participants making hypothetical decisions between receiving immediate small monetary amounts or waiting for a fixed delay to receive EUR 10 (e.g., a sample question might inquire whether the participant prefers EUR 2 now or EUR 10 in 30 days). An algorithm iteratively adjusts the immediate amount until the participant becomes indifferent between the immediate and delayed options, employing a random adjusting procedure [66]. This indifference point, determined for each participant, signifies the subjective value assigned to the delayed larger reward relative to an immediate sum [66]. The task encompasses five delay intervals (0, 10, 30, 180, and 365 days) to assess delay discounting. Following the methodology outlined in [67], indifference points are utilized to estimate delay discounting. Indifference points are then normalized as proportions of the maximum delayed reward (EUR 10), and delays are normalized as proportions of the maximum delay (365 days). Normalized values are used to construct a delay discounting plot, with delay and indifference points represented on the x and y axes, respectively. Each data point yields four distinct trapezoids on the x axis, whose areas are calculated using the formula (x_2_ − x_1_) · [(y_1_ + y_2_)/2]. The areas under these trapezoids represent the overall delay discounting (Area Under the Curve (AUC)), ranging from 0 (maximum discounting) to 1 (no discounting). Higher AUC scores indicate lesser impulsivity, reflecting reduced discounting by delay. Delay discounting demonstrates good test–retest reliabilities, with documented correlation coefficients of rs= 0.67 and 0.76, respectively [68].

### 2.3. Clinical Tools

#### 2.3.1. Melbourne Assessment of Schizotypy in Kids (MASK)

The MASK is a structured assessment tool designed to evaluate features of schizotypal disorder in children aged 5 to 12 years [69]. Comprising 57 items, it encompasses nine domains: social anxiety, social skills, motor abilities, language/thought/ideation, fantasy/magical thinking, unusual perceptual experiences, behavior, attention, and affect. MASK scores range from 57 to 228 (cutoff score of 132 as indicative of schizotypy) and demonstrate robust internal consistency (Cronbach’s α = 0.98), high inter-rater reliability (0.98) [50], and good convergent validity with established measures such as BASC II [70] and the CRS-R [71]. Furthermore, the MASK exhibits excellent diagnostic utility in discriminating between ASD and schizotypy [69]. The MASK’s factorial structure comprises two factors: Factor 1, relating to socio-pragmatic skills, and Factor 2, reflecting positive schizotypal symptoms in children [69].

#### 2.3.2. Autism Diagnostic Observation Schedule—2nd Edition (ADOS-2 [72])

The ADOS-2 comprises five modules tailored to individuals with diverse expressive language and developmental levels. Modules 1 and the Toddler Module are aimed at children who do not consistently use phrase speech, whereas Module 2 targets those who use phrase speech but are not yet verbally fluent. Modules 3 and 4 are intended for individuals with fluent speech, encompassing children and adolescents who engage in appropriate play with action figures and older adolescents and adults, respectively. The diagnostic algorithms of the ADOS-2 yield domain scores for social affect and restricted and repetitive behaviors, alongside a total score. Modules 1 to 4 offer three diagnostic classifications: non-spectrum, Autism spectrum disorder (ASD), and Autism. The Toddler Module presents ranges of concern, classifying observed behaviors as having little-to-no concern, mild-to-moderate concern, or moderate-to-severe concern.

#### 2.3.3. Procedure

All neuropsychological tests were administered individually in a quiet, distraction-free room within the Special ASD clinic. Assessments were conducted by experienced child psychiatrists and psychologists, who followed a standardized protocol to ensure consistency across participants. Before beginning, children were given a short familiarization period to establish rapport and reduce test anxiety. The tasks were administered in a counterbalanced order across participants to control for order effects. Short breaks were provided between tasks to mitigate fatigue, particularly for longer assessments.

#### 2.3.4. Statistical Analysis

Analyses were conducted using SPSS statistical software (version 22.0). Variables were first tested for normality using the Kolmogorov–Smirnov criterion. Spearman correlations coefficients were used to explore the association between ADOS-2 (total score and subscales), MASK (total score and subscales), and EF tests. In order to adjust for children’s age and gender, partial correlation coefficients were computed. Finally, we conducted a series of hierarchical regression analyses in order to investigate the effects of the co-occurrence of ADOS and MASK scores on all cool and hot EF tests. The predictors included in the analysis were age, gender, MASK total score, ADOS total score, and the interaction between MASK and ADOS total scores. The dependent variables comprised the following cool and hot EF tests: Stroop, ToL, Digit Span, BCST, Delay Discounting, and IGT. Control variables (age and gender) were entered in Block 1, MASK and ADOS total scores were entered in Block 2, and finally the MASK × ADOS interaction term was entered in Block 3 (centered to reduce multicollinearity). All tests were two-tailed, and statistical significance was set at *p* < 0.05.

## 3. Results

### 3.1. Correlational Analysis

Table 1 presents the sample’s characteristics and the results of children’s EF tests, alongside their scores on the ADOS and MASK scales.

The correlations between the MASK scale and EF tests are detailed in Table 2. A significant negative correlation was observed between the Digit Span score and the total MASK score. Even after adjusting for children’s age and gender, this association remained significant (r_partial = −0.32, *p* = 0.048). It should be noted that after applying Benjamini–Hochberg correction for multiple comparisons, all correlations dropped to non-significance.

Additionally, Table 2 displays associations between ADOS scores and EF tests. A higher value in the Stroop test was significantly associated with a greater score in the Restricted and Repetitive Behavior subscale. This association remained significant even after adjusting for children’s age and gender (r_partial = 0.36, *p* = 0.005). Furthermore, a greater Digit Span score was associated with a lower score in the Social Affect subscale. However, after adjusting for children’s age and gender, this association became indicative (r_partial = −0.23, *p* = 0.073). Moreover, a higher ToL score was significantly associated with a lower score in the Social Affect subscale, as well as with a lower total score. These correlations remained significant after adjusting for children’s age and gender (r_partial =−0.28, *p* = 0.032 and r_partial =−0.25, *p* = 0.049, respectively). It should be noted that after applying Benjamini–Hochberg correction for multiple comparisons, all correlations—except for the association between Stroop and ADOS Restricted and Repetitive Behavior (original *p* = 0.002/adjusted *p* = 0.03)—dropped to non-significance.

### 3.2. Hierarchical Regression Analysis

Table 3 shows that for the first five models (all assessing cool EF tests and the hot Delay Discounting task), there was no significant ADOS × MASK interaction found.

With regard to model 6 (hot IGT test), the first block (age and gender) [*F* (2,60) = 0.66, *p* = 0.52] as well as the second one (MASK and ADOS scores) [*F* (2,58) = 0.92, *p* = 0.41] did not contribute significantly to the variance of the IGT test. However, the interaction ADOS × MASK term, introduced in block 3, explained 9.8% [*F* (1,57) = 8.2, *p* = 0.006)] of the variance. The IGT test was significantly predicted by the interaction ADOS × MASK term (*p* = 0.006). This interaction suggests that the relationship between the level of autism-related symptoms (measured by ADOS) and schizotypal traits (measured by MASK) significantly influences affective decision-making as assessed by the IGT. The negative coefficient (−0.35) suggests that higher levels of combined autism-related symptoms and schizotypal traits are associated with poorer performance on the IGT. After applying the Benjamini–Hochberg (B-H) correction for multiple comparisons in the hierarchical regression analyses, the interaction effect remained significant (*adjusted p* = 0.03), confirming the robustness of this association.

## 4. Discussion

The present study aimed to investigate the role of the ASD and schizotypal traits’ co-occurrence on cool and hot EF in children with ASD. The findings reveal significant associations between selective EF aspects and specific ADOS subscales, while MASK was only significantly related to cool working memory. Finally, the hierarchical regression model analysis revealed that the interaction between the MASK total score and the ADOS total score, reflecting the co-occurrence of ASD and schizotypal traits, significantly predicted hot EF affective decision-making (as measured by IGT scores). These findings have important implications for understanding the cognitive profiles of children with ASD and schizotypal traits.

### 4.1. MASK and EF Relation

A significant negative correlation was found between the Digit Span score and the total MASK score, even after adjusting for age and gender. This suggests that children with higher schizotypal traits, as measured by the MASK, may struggle with tasks requiring working memory. Dysfunctions in working memory are widely recognized as fundamental features of schizophrenia spectrum disorder [18,73,74]. Notably, working memory has been proposed as a potential endophenotypic marker for the disorder [18]. Subtle impairments in working memory have been also documented in association with elevated levels of positive [75] and negative [76] schizotypy. It has been suggested that working memory deficits may signify a diminished accuracy in downstream information processing, which could be evident even in early electrophysiological measures [77]. Fronto-temporal brain circuits are integral to working memory processes (e.g., [78]), while morphological and functional changes in these circuits have been most frequently associated with schizotypy (for a review, see [79]). Disruptions within these neural networks may compromise the efficient encoding, maintenance, and retrieval of information, leading to impairments in working memory performance among individuals with elevated schizotypal traits. While our findings suggest potential associations between schizotypal traits and EF, these results should be interpreted with caution, as statistical corrections for multiple comparisons reduced all associations to non-significance. Nevertheless, as the observed patterns align with prior research, they underscore the need for further investigations on larger samples with stricter statistical controls.

### 4.2. ADOS and EF Relation

A higher Stroop test score, indicative of poorer inhibition, was associated with a greater score in the Restricted and Repetitive Behavior subscale of ADOS. This finding aligns with those in the literature suggesting that individuals with ASD often exhibit difficulties with inhibition (see [27] for a meta-analysis). These findings are in line with previous evidence reporting that heightened levels of restricted and repetitive behaviors were related to reduced inhibition in ASD (e.g., [80]). Inhibition relates to the severity of these symptoms in ASD as the inability to inhibit prepotent responses or ignore irrelevant information could result in the perseveration and rigid adherence to routines or patterns characteristic of repetitive behaviors in ASD. Inhibition deficits could exacerbate the sensory processing challenges faced by children with ASD, by impairing the ability to filter out irrelevant or distracting sensory information. As a result, children with ASD may engage in repetitive and restricted behaviors as a way to cope with or regulate their sensory experiences.

Furthermore, a greater Digit Span score was associated with a lower score in the Social Affect subscale, but this association became indicative after adjusting for age and gender, suggesting that better working memory performance may be linked to fewer social affect difficulties in ASD. Working memory is responsible for temporarily storing and manipulating information, which is crucial for social interactions. Children with ASD with better working memory skills may exhibit more efficient cognitive processing during social interactions. They may be better able to retain and process social cues, leading to improved understanding and response in social situations. As a result, they may experience fewer difficulties in interpreting social cues, understanding social norms, maintaining eye contact, recognizing facial expressions, and understanding gestures (as assessed in the Social Affect subscale) compared to those with lower working memory capacity.

Finally, a greater ToL score was significantly associated with a lower score in the Social Affect subscale, as well as with a lower total ADOS score. These correlations remained significant after adjusting for age and gender, suggesting that better planning and problem-solving skills may be associated with fewer ASD traits. Planning and problem-solving skills are crucial components of adaptive social functioning. Effective planning allows individuals to anticipate social situations, make appropriate decisions, and adjust their behavior accordingly. Conversely, lower performance in planning may lead to difficulties in navigating social interactions and understanding social cues in ASD.

Similar to the MASK-EF relation, the results regarding the associations between ADOS scores and EF performance should be interpreted with caution, as statistical corrections for multiple comparisons reduced all but one association (Stroop and ADOS Restricted and Repetitive Behavior) to non-significance. However, the observed patterns remain consistent with those in prior research, highlighting the potential role of executive dysfunction in ASD symptomatology. Future studies with larger samples and stricter statistical controls are needed to further clarify these relationships.

### 4.3. ADOS × MASK Interaction

The results of the hierarchical regression model analysis show that the interaction between the MASK total score and the ADOS total score significantly affected the hot EF task IGT. This suggests that the relationship between the level of co-occurrent autism-related symptoms and schizotypal traits significantly influences affective decision-making. The negative coefficient implies that higher levels of autism-related symptoms combined with higher schizotypal traits may lead to poorer affective decision-making. The interaction effect remained significant after applying the Benjamini–Hochberg correction, indicating that the observed association is statistically robust.

This finding appears to contradict previous research (e.g., [2]) suggesting that the comorbidity of ASD and schizotypal traits can alleviate EF difficulties. One potential explanation for this inconsistency might stem from the particular EF domains under examination. Previous research [49] solely concentrated on cool EF attentional set shifting when exploring this correlation, whereas we comprehensively investigated both cool and hot EF aspects in our study. It is likely that the comorbidity of ASD and schizotypal traits may have differential effects on cool and hot EF domains, and while the co-occurrence of ASD and schizotypal traits may improve some selective cool EF aspects, hot EFs might be more sensitive to the negative effects of this comorbidity. The interaction of schizotypal and autistic traits seems to have a more complex and nuanced impact on hot EF, possibly due to the emotional/motivational nature of these processes.

Another plausible explanation for this could be related to the cognitive style associated with ASD, characterized by a tendency towards detail-focused processing and adherence to rules. Schizotypal traits, which may involve cognitive flexibility and unconventional thinking, could potentially counterbalance some of the rigid cognitive patterns associated with ASD, thereby improving performance on cool EF tasks. However, when considering hot EF domains, the interaction between ASD and schizotypal traits might have a different impact. Hot EF tasks, such as the IGT, involve emotionally salient stimuli and require integrating cognitive processes with affective and motivational/reward components. Schizotypal traits, which are associated (among others) with unusual perceptual experiences, eccentric behavior, and social anhedonia, may disrupt the integration of emotional and cognitive processes necessary for effective decision-making in such contexts. In children with ASD, who already face challenges in understanding and regulating emotions, the addition of schizotypal traits may further exacerbate difficulties in processing emotional/motivational information and making adaptive decisions. Understanding how specific combinations of traits affect cognitive processes, especially in emotionally charged decision-making situations, may lead to tailored interventions and support strategies for individuals with these traits.

### 4.4. Limitations

While the present study contributes valuable insights into the cognitive profiles of children with ASD and schizotypal traits, several limitations warrant consideration. A potential concern in assessing schizotypal traits in children is the developmental stability of personality disorders at this age. Personality is still evolving during childhood and adolescence, and most personality disorders are usually diagnosed in adulthood. However, dimensional approaches to schizotypy emphasize the assessment of traits rather than categorical diagnosis. Studies have shown that early schizotypal traits can serve as precursors to later schizotypal symptomatology (e.g., [8]), reinforcing the importance of studying schizotypy in childhood through a neurodevelopmental lens. Moreover, a notable limitation of the study pertains to the absence of distinct groups, which precluded comparisons with groups of controls, ASD only, schizotypy only, and co-occurring ASD and schizotypy. Distinct groups would have allowed for a more rigorous examination of the specificity of the observed associations between ASD symptoms, schizotypal traits, and EF domains. Moreover, the study involved a relatively small sample size, which may limit the statistical power to detect subtle effects or interactions, particularly when exploring complex relationships between multiple variables. Another limitation of the present study is the potential inflation of significance levels due to multiple comparisons. Although our analyses were hypothesis-driven and guided by prior literature, applying Benjamini–Hochberg correction led to most correlations falling below the significance threshold. While this underscores the importance of cautious interpretation, the observed patterns remain consistent with previous research. Future studies with larger samples and enhanced statistical power are needed to further confirm and refine these findings. Finally, the cross-sectional nature of the study design limits the ability to establish causal relationships between variables. Future longitudinal studies are needed to elucidate the developmental trajectories of these cognitive processes over time, and to determine the link of ASD symptoms and schizotypal traits to EF difficulties.

## 5. Conclusions

This study examined the impacts of co-occurring ASD and schizotypal traits on hot and cool EF in children with ASD. Our findings demonstrate that while ASD and schizotypal traits are both associated with EF difficulties, their co-occurrence uniquely impacts affective decision-making (hot EF), as evidenced by the significant ADOS × MASK interaction predicting Iowa Gambling Task performance. Importantly, this effect remained significant even after statistical correction, reinforcing its robustness. These results contribute to the growing body of literature on the cognitive correlates of ASD and schizotypal traits, offering new insights into how their combined effects may affect motivationally salient cognitive processes.

Future studies should aim to replicate these findings in larger and more diverse samples, employing a broader range of EF domains, to further clarify whether the observed patterns are consistent across different developmental stages. Additionally, investigating the neural correlates of hot EF in ASD-schizotypy co-occurrence could provide further insights into these cognitive differences. This will help to provide a more nuanced understanding of the cognitive profiles of individuals with ASD and schizotypal traits, and inform the development of targeted interventions to support these individuals. Given that co-occurring ASD and schizotypal traits were associated with poorer affective decision-making, interventions aimed at improving hot EF (e.g., affective decision-making, delay discounting skills) could be more heavily emphasized for children presenting with these traits. Programs incorporating reinforcement-based learning strategies, real-life decision-making scenarios, and emotion-based cognitive training may help improve real-world adaptive functioning for these children.

## Figures and Tables

**Table 1:** Sample’s characteristics and scores in EF tests, ADOS, and MASK scales.

*Child’s Sex*	
Males N (%)	51 (81.0)
Females N (%)	12 (19.0)
*Child’s age*, mean (SD)	9.3 (1.9)
*EF test*	Mean (SD)
Stroop Test	6.9 (6.16)
Digit Span	15.24 (3.96)
ToL	7.11 (1.93)
BSCT	43.08 (8.79)
IGT	−0.03 (0.17)
Delay Discounting	0.43 (0.24)
*ADOS-2*	
Social Affect	12.3 (4.57)
Restricted and repetitive behavior	2.86 (1.82)
Total ADOS-2 score	15.16 (5.67)
*Mask*	
Total MASK score	135.81 (21.82)
Social/Pragmatic symptoms	83.44 (10.28)
Positive schizotypal symptoms	53.25 (15.56)

**Table 2:** Spearman’s correlation coefficients between MASK scale, ADOS scale, and neuropsychological tests.

		Total MASK Score	Social/Pragmatic Symptoms	Positive Schizotypal Symptoms	ADOS Social Affect	ADOS Restricted and Repetitive Behavior	Total ADOS Score
**Stroop Test**	rho	0.01	0.12	−0.11	0.10	0.40	0.20
	*p*	0.938	0.361	0.388	0.465	0.002	0.123
**Digit Span**	rho	−0.25	−0.19	−0.19	−0.25	−0.02	−0.18
	*p*	0.047	0.126	0.127	0.050	0.898	0.150
**ToL**	rho	−0.06	−0.06	−0.06	−0.26	−0.08	−0.25
	*p*	0.633	0.64	0.656	0.042	0.534	0.050
**BSCT**	rho	−0.18	−0.09	−0.21	−0.19	−0.03	−0.17
	*p*	0.153	0.462	0.098	0.128	0.816	0.193
**IGT**	rho	0.13	0.13	0.09	0.01	−0.03	−0.02
	*p*	0.302	0.328	0.479	0.915	0.845	0.897
**Delay Discounting**	rho	−0.09	0.03	−0.12	0.03	0.15	0.08
	*p*	0.494	0.813	0.354	0.829	0.270	0.529

**Table 3:** Hierarchical regression analysis.

Block	Predictors	Dependent Variables											
		**Stroop**		**ToL**		**Digit Span**		**BCST**		**Delay Discounting**		**IGT**	
		**B**	***p***	**B**	***p***	**B**	***p***	**B**	***p***	**B**	***p***	**B**	***p***
**1**	**Age** **Gender**	−0.5 −0.13	0.25 0.52	0.3 −0.57	0.02 0.34	0.76 −0.13	0.005 0.91	1.12 0.66	0.07 0.81	0.01 0.05	0.41 0.54	−0.13 0.06	0.30 0.62
**2**	**MASK** **total score** **ADOS** **total score**	−0.02 0.21	0.61 0.18	0.01 −0.07	0.57 0.13	−0.02 −0.04	0.48 0.67	−0.02 −0.15	0.76 0.47	−0.001 0.01	0.51 0.16	0.18 −0.02	0.19 0.89
**3**	**MASK** × ADOS **Interaction**	−0.001	0.87	0.01	0.92	0.01	0.14	−0.004	0.72	0.07	0.61	−0.35	0.006

## Data Availability

The data presented in this study are available on request from the corresponding author due to ethical reasons.
